# Impact of CD151 overexpression on prognosis and therapy in non‐small cell lung cancer patients lacking EGFR mutations

**DOI:** 10.1111/cpr.13708

**Published:** 2024-07-09

**Authors:** Amanda Huee‐Ping Wong, Min En Nga, Chin Yein Chin, Yee Kit Tai, Hung Chew Wong, Ross Soo, Omer An, Henry Yang, Ju Ee Seet, Yaw Chyn Lim, John Kit Chung Tam, Thai Tran

**Affiliations:** ^1^ Department of Physiology, Yong Loo Lin School of Medicine National University of Singapore Singapore Singapore; ^2^ Department of Pathology National University Hospital Singapore Singapore; ^3^ Department of Pathology, Yong Loo Lin School of Medicine National University of Singapore Singapore Singapore; ^4^ Department of Biostatistics, Yong Loo Lin School of Medicine National University of Singapore Singapore Singapore; ^5^ Department of Haematology‐Oncology National University Hospital Singapore Singapore; ^6^ Cancer Science Institute of Singapore National University of Singapore Singapore Singapore; ^7^ Department of Surgery, Yong Loo Lin School of Medicine National University of Singapore Singapore Singapore; ^8^ Department of Cardiac, Thoracic and Vascular Surgery, National University Heart Centre, Singapore National University Health System Singapore Singapore; ^9^ Infectious Disease Translational Research Programme, Yong Loo Lin School of Medicine National University of Singapore Singapore Singapore

## Abstract

This study investigates CD151, a protein linked to cancer progression, in non‐small cell lung cancer (NSCLC) patients without epidermal growth factor receptor (EGFR) mutations. These patients often have limited treatment options. The study used retrospective analysis to examine 157 adenocarcinoma biopsy specimens and 199 patient cases from The Cancer Genome Atlas, correlating CD151 expression with patient survival. Cellular studies revealed that CD151 interacts with EGFR, influencing epidermal growth factor (EGF)‐induced cell proliferation and the effectiveness of the EGFR inhibitor, erlotinib. A strong association was found between CD151 expression and EGFR mutation status. High CD151 expression in the absence of EGFR mutations is correlated with poorer survival outcomes. Biological assays showed that CD151 colocalizes and associates with EGFR, playing a crucial role in regulating EGF‐induced cell proliferation via the AKT and ERK1/2 pathways. Importantly, CD151 expression was found to influence the anti‐proliferative effects of the EGFR tyrosine kinase inhibitor, erlotinib. High CD151 expression, in the absence of EGFR mutations, was associated with poorer survival outcomes. It could serve as a potential prognostic marker and influence cellular responses to EGFR‐targeted treatments. This study highlights CD151 as a potential novel target for therapeutic intervention in NSCLC, especially in populations lacking EGFR mutations.

## Graphical abstract

High CD151 expression levels in non‐small cell lung cancer (NSCLC) patients lacking epidermal growth factor receptor (EGFR) mutations are associated with poorer clinical outcomes. Mechanistically, CD151 influences Akt‐ERK‐dependent cell growth and the effectiveness of EGFR tyrosine kinase inhibitors. Our study proposes CD151 as a potential therapeutic target in NSCLC patients without EGFR mutations whose treatment options are currently limited.

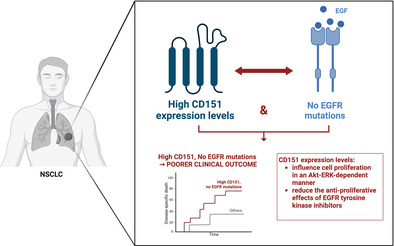

## 1 INTRODUCTION

Lung cancer remains the malignancy with the highest mortality rate globally and the poorest survival outlook,^1^, ^2^ for which non‐small cell lung cancer (NSCLC) accounts for 85% of lung cancer cases.^3^ Poor survival may be attributed to the underutilization of early diagnostic tests and limited treatment options.^4^, ^5^ Despite newly developed molecularly targeted therapy and immunotherapy, a subset of patients do not benefit from these novel therapeutics due to a lack of actionable biomarkers^6^, ^7^ or low expression of favourable predictive biomarkers.^5^, ^8^ These considerations, along with the underwhelming efficacy of molecular therapy in early‐stage patients and immune‐related adverse events with immunotherapy,^5^ restrict drastic improvements in disease management and encourage the identification of novel therapeutic targets.

One potential target is the epidermal growth factor receptor (EGFR), which is frequently mutated and activated in NSCLC, promoting tumour growth and survival.^9^ Despite the favourable clinical responses ascribed to molecular targeted therapy (such as anti‐EGFR tyrosine kinase inhibitors [TKIs] or monoclonal antibodies), clinical benefit is largely restricted to NSCLC patients with classical activating EGFR mutations,^10^, ^11^ namely, exon 19 and exon 21 L858R mutations. The global frequencies of these classical EGFR mutations in lung cancer and NSCLC patients were reported as 17% and 33%, respectively.^12^, ^13^ The primary treatment option for patients who lack these EGFR mutations is restricted to platinum‐based chemotherapy with or without immunotherapy,^14^, ^15^ for which adverse effects and acquired resistance pose significant management challenges. Whilst osimertinib and targeted therapy for mutations such as anaplastic lymphoma kinase (ALK) and ROS1 have demonstrated some clinical benefit in patients lacking these EGFR mutations,^16^, ^17^ they do not fully address the broader NSCLC population without these mutations. The ongoing difficulties in managing the shortcomings of existing treatments highlight the urgent need to investigate new therapeutic strategies. This is to improve patient outcomes and effectively address the limitations of current treatment options.

Another possible target is the tetraspanin, CD151, a protein that interacts with EGFR^18^ and other receptors to regulate various cellular functions.^19^ Overexpression of CD151 and its negative prognostic impact have been documented in breast, prostate and liver cancer.^20^, ^21^, ^22^, ^23^ The dysregulation of multiple human malignancies may be attributed to the broad distribution of CD151, especially in the lungs.^24^ Unsurprisingly, elevated CD151 expression was linked to poorer clinical outcomes in NSCLC^25^, ^26^ whilst preclinical studies have reported the role of CD151 as a tumour promoter in NSCLC.^19^ Despite existing evidence on the influence of CD151 expression and the presence of EGFR mutations in NSCLC separately, a significant gap exists in understanding the landscape of NSCLC in the absence of these EGFR mutations. In this study, we aim to fill this knowledge gap by investigating the clinical and functional impact of CD151 expression in two cohorts of adenocarcinoma patients and NSCLC models without EGFR mutations.

## 2 METHODS

### 2.1 Study cohorts

Retrospective analysis of 157 patient biopsy specimens was obtained from adenocarcinoma patients at the National University Hospital (NUH), Singapore (inclusion criteria found in Figure S1). The median age of patients was 63 years; 94 (62%) patients were male, and 113 (75%) patients were of Chinese ethnicity. Seventy‐nine (53%) patients had no EGFR mutations, whereas 69 (47%) patients had one or more EGFR mutations (Table S1). Clinical data of 199 NSCLC cases without EGFR mutations from The Cancer Genome Atlas (TCGA) lung adenocarcinoma (LUAD) cohort was obtained for survival analysis based on CD151 expression. This study population, comprised of 90 (45%) male patients, was predominantly Caucasian (191/199 patients, 96%) (Table S2). The samples and data were obtained with approval from the National Healthcare Group DSRB ethics committee (2015/00099‐SRF0002).

### 2.2 Tissue preparation and immunohistochemical analysis

**FIGURE 1:**
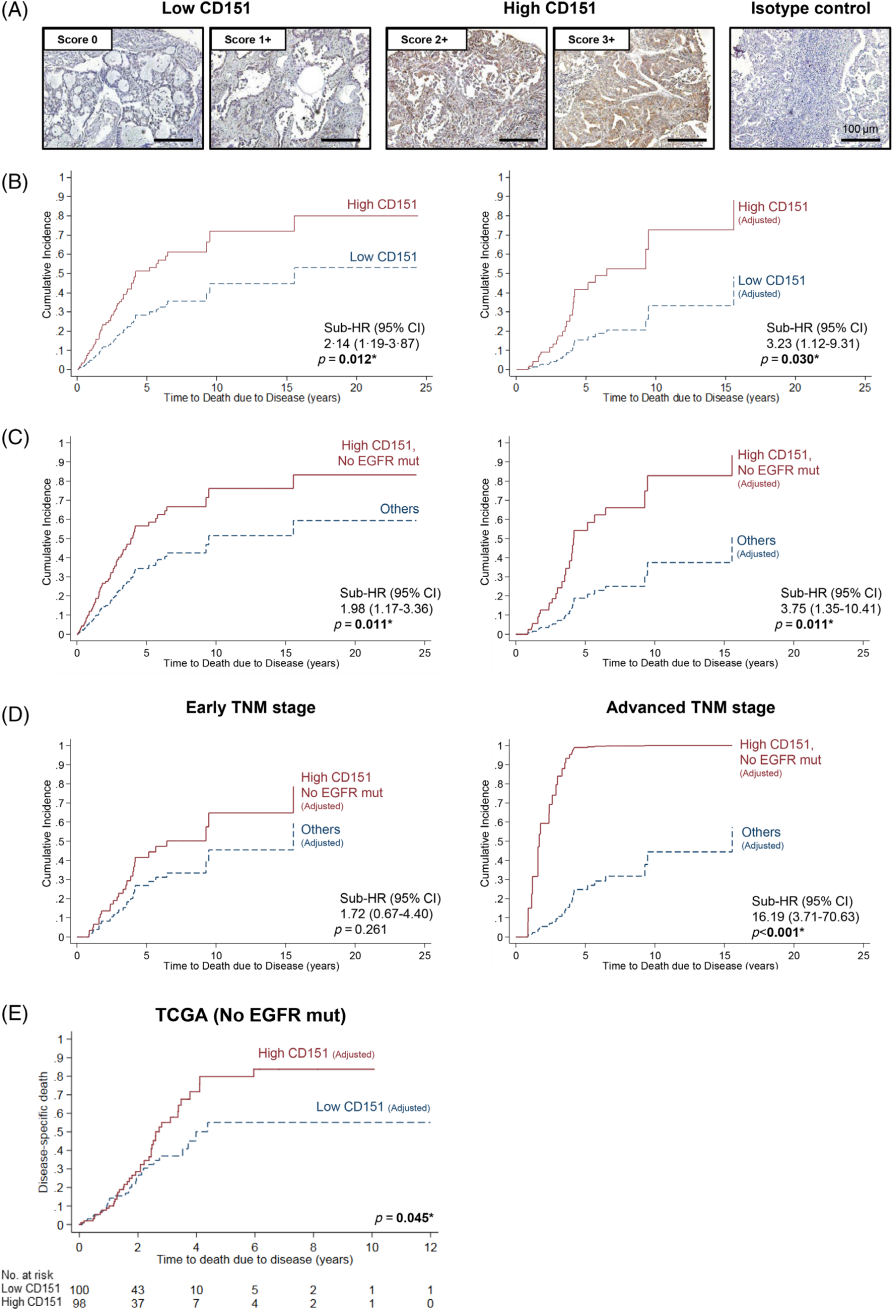
Non‐small cell lung cancer (NSCLC) patients with high CD151 and no epidermal growth factor receptor mutation (EGFR mut) tumours had a poorer survival outcome. (A) Images of representative immunohistochemical analyses of CD151 expression in lung tissue biopsy sections from the National University Hospital (NUH) Singapore cohort for each intensity and corresponding isotype control. All scale bars represent 100 μm. (B) Unadjusted and adjusted cumulative incidence of high CD151 versus low CD151 patients. (C) Unadjusted and adjusted cumulative incidence of high CD151 patients with no EFGR mut versus others and (D) adjusted cumulative incidence of this subgroup following stratification according to tumour, node, metastasis (TNM; American Joint Committee on Cancer [AJCC]) stage. (E) Disease‐specific death of high CD151 versus low CD151 in patient cases without EGFR mut from The Cancer Genome Atlas (TCGA) lung adenocarcinoma cohort. *p < 0.05. Survival analysis differences were analysed using competing‐risks regression (NUH) and log‐rank test (TCGA) respectively. CI, confidence interval; Sub‐HR, subhazard ratio.

Tissue microarrays of formalin‐fixed paraffin‐embedded tissue blocks obtained from NUH were generated according to methods previously described and validated.^27^ Four‐micron‐thick microarray tissue sections were stained with CD151 antibody (Clone 115GA, Bio‐Rad) and scored according to a 4‐tier classification system (Figure 1A) as previously described^25^, ^28^ in a blinded fashion by two independent observers with good inter‐observer agreement (*κ* = 0.91).

### 2.3 EGFR mutation analysis

Mutation analysis for the NUH cohort was performed for EGFR exons 18, 19, 20 and 21 via Sanger sequencing according to established protocols.^29^

### 2.4 Immunofluorescence and measurement of mean cell fluorescence and colocalization

Immunofluorescence was performed as previously described^30^ for CD151 and EGFR (Cell Signaling Technology, CST). Fluorescence was imaged with the Olympus FluoView FV10i laser scanning confocal microscope. ImageJ software was used to determine mean cell fluorescence and colocalization (detailed methods in Supporting Information).

### 2.5 Co‐immunoprecipitation

Cell lysates were collected using three different lysis buffers: Triton‐X 100, Brij‐97 and 3‐[(3‐Cholamidopropyl)dimethylammonio]‐1‐propanesulfonate CHAPS hydrate (Sigma‐Aldrich). Immunoprecipitation was conducted by combining CD151 or EGFR‐bound protein G Sepharose beads with 300 μg of cell lysates. Samples were washed and resuspended in loading buffer for immunoblotting.

### 2.6 Immunoblotting

Immunoblotting of protein lysates was carried out as previously described^30^ using the following antibodies: CD151 (BioRad), Glyceraldehyde 3‐phosphate dehydrogenase (GAPDH) (Santa Cruz Biotechnology), Integrin α3 (ITGA3, Abcam), EGFR, pAKT (phosphorylated AKT, S473), AKT, phosphorylated extracellular signal‐regulated kinase (pERK1/2) (T202/Y204) and ERK1/2 (CST).

### 2.7 Cell culture and reagents

A549 cells (American Type Culture Collection) were cultured in complete Dulbecco’s Modified Eagle’s Medium (Gibco). Two commercially available CD151 small interfering RNA (siRNA) kits were purchased from Dharmacon and Qiagen to knockdown CD151 protein expression according to the manufacturer's instructions. Conversely, stably CD151‐overexpressing (CD151 OE) and corresponding vector control A549 cells were generated using pEGFP‐CD151 or pEGFP‐N3 (empty) vectors, respectively (detailed methods in Supporting Information).

### 2.8 Cell enumeration

Cells were counted by the trypan blue exclusion method, as previously described.^31^

### 2.9 Statistical analysis

Clinical data and in vitro experiments were statistically analysed using Stata (v13.1) and GraphPad Prism 6 softwares, respectively, according to relevant analyses where *p* < 0.05 was deemed statistically significant.

## 3 RESULTS

### 3.1 CD151 overexpression predicts poor survival in lung cancer patients without EGFR mutations

To explore the clinical significance of CD151 expression in NSCLC, CD151 expression levels were first analysed via immunohistochemical staining of tissue biopsies from 155 NSCLC patients in the NUH cohort (Figure 1A). We showed that 102 patients had high levels of CD151 expression. High CD151 expression was associated with several factors: ethnicity (*p* = 0.048), tumour, node, metastasis (TNM; American Joint Committee on Cancer [AJCC]) stage, which is a measure of cancer progression (*p* = 0.019), and smoker status (*p* = 0.022). Further investigation into the smoker status revealed that smokers were significantly more likely to express high levels of CD151 compared to non‐smokers. The odds ratio 4.14, with a 95% confidence interval (CI) of 1.11–15.48 (*p* = 0.032). For the first time, this study reports an association between high CD151 expression and EGFR mutation status (*p* = 0.030). Specifically, high CD151 expression was observed in 77% of patients whose tumours did not have EGFR mutations (Table 1).

**TABLE 1:** Relationship between CD151 expression and clinical factors in the Singaporean cohort.

	CD151 expression		
	Low (*n* = 53)	High (*n* = 102)	*p* Value
Mean age, SD (years)	63.49 (9.30)	61.61 (10·38)	0.157
Sex			0.379
Male	29 (31.5)	63 (68.5)	
Female	23 (39.7)	35 (60.3)	
Ethnicity			**0.048** *
Chinese	34 (30.1)	79 (69.9)	
Others	18 (48.6)	19 (51.4)	
Smoking history			**0.022** *
Non‐smoker	25 (41.5)	35 (58.3)	
Ex‐smoker	8 (34.8)	15 (65.2)	
Smoker	5 (14.7)	29 (85.3)	
TNM stage			**0.019** *
I	38 (44.7)	47 (55.3)	
II	4 (18.2)	18 (81.8)	
III	4 (20.0)	16 (80.0)	
IV	3 (18.8)	13 (81.2)	
pT stage			0.205
T1	26 (40.6)	38 (59.4)	
T2a	18 (32.7)	37 (67.3)	
T2b	1 (12.5)	7 (87.5)	
T3	1 (11.1)	8 (88.9)	
Tumour grade			0.101
WD	6 (54.5)	5 (45.5)	
MD	33 (34.7)	62 (65.3)	
PD	5 (19.2)	21 (80.8)	
EGFR subtype			**0.005** *
No EGFR mut	18 (23.4)	59 (76.6)	
EGFR mut	32 (46.4)	37 (53.6)	

*Note*: Data are number (%) unless otherwise stated. Bold indicates siginificant value (*p* < 0.05).

Abbreviations: EGFR, epidermal growth factor receptor; EGFR mut, EGFR mutation; MD, moderately differentiated; PD, poorly differentiated; pT, primary tumour; TNM, tumour, node, metastasis (American Joint Committee on Cancer [AJCC]) stage; WD, well‐differentiated.

* *p* < 0.05, as calculated by Fisher's exact test.

Given the novel association of CD151 overexpression and EGFR mutation status, we next examined the clinical significance of this association by analysing survival data from the NUH cohort. We found that patients with high levels of CD151 had a shorter survival time than those with low levels of CD151, including after adjustment for other confounding factors such as age, gender and smoking status (subhazard ratio [Sub‐HR], 95% CI 3.23 [1.12–9.31], *p* = 0.030; Figure 1B; Table 2). We then divided the patients into four subgroups based on their CD151 levels and whether they had mutations in the EGFR gene, which is often altered in lung cancer. We found that the subgroup with high CD151 and no EGFR mutations had the worst survival outcome compared to the other subgroups (Sub‐HR [95% CI] 3.75 [1.35–10.41], *p* = 0.011; Figure 1C, Table 3). This subgroup had a higher risk of dying from lung cancer at any given time point, such as 1, 5 or 10 years after diagnosis (1‐year 2.5% vs. 0.6%; 5‐year 54.4% vs. 19.4%; 10‐year 82.5% vs. 37.5%).

**TABLE 2:** Competing‐risks regression of time to death due to disease in Singaporean cohort.

	Cumulative incidence	
	Subhazard ratio (95% CI)	*p* value
Age	1.05 (1.00–1.11)	**0.049** *
Sex		
Female	1.00	—
Male	0.95 (0.40–2.26)	0.904
Ethnicity		
Chinese	1.00	—
Others	0.18 (0.03–1.09)	0.602
Smoking history		
Non‐smoker	1.00	—
Ex‐smoker	0.60 (0.17–2.16)	0.436
Smoker	0.66 (0.24–1.81)	0.422
TNM stage		
I, II	1.00	—
III, IV	5.73 (1.75–18.73)	**0.004** *
Tumour stage		
T1, T2a	1.00	—
T2b, T3	0.50 (0.13–1.89)	0.305
Tumour grade		
WD	1.00	—
MD	0.91 (0.37–2.25)	0.840
PD	1.42 (0.42–4.72)	0.572
Tumour stage		
T1, T2a	1.00	—
T2b, T3	1.83 (0.60–5.58)	0.289
CD151 expression		
Low CD151	1.00	—
High CD151	3.23 (1.12–9.31)	**0.030** *

*Note*: Reference factors are stated first. Bold indicates siginificant value (*p* < 0.05).

Abbreviations: CI, confidence interval; MD, moderately differentiated; PD, poorly differentiated; TNM, tumour, node, metastasis (American Joint Committee on Cancer [AJCC]) stage; WD, well‐differentiated.

* *p* < 0.05 as calculated by competing‐risks regression.

**TABLE 3:** Competing‐risks regression of time to death due to disease for high CD151, no epidermal growth factor receptor (EGFR) mutations group versus others in the Singaporean cohort.

	Cumulative incidence	
	Subhazard ratio (95% CI)	*p* Value
Age	1.04 (0.99–1.09)	0.100
Sex		
Female	1	—
Male	0.93 (0.40–2.17)	0.864
Ethnicity		
Chinese	1	—
Others	0.17 (0.03–0.85)	**0.031** *
Smoking history		
Non‐smoker	1	—
Ex‐smoker	0.71 (0.20–2.50)	0.599
Smoker	0.62 (0.23–1.69)	0.353
TNM stage		
I, II	1	—
III, IV	6.70 (1.94–23.12)	**0.003** *
Tumour stage		
T1, T2a	1	—
T2b, T3	0.42 (0.11–1.63)	0.210
Tumour grade		
WD	1	—
MD	0.96 (0/36–2.54)	0.930
PD	1.62 (0.44–6.05)	0.471
Subgroup		
Others	1.00	—
High CD151, no EGFR mut	3.75 (1.35–10.41)	**0.011** *

*Note*: Reference factors are stated first. Bold indicates siginificant value (*p* < 0.05).

Abbreviations: CI, confidence interval; EGFR mut, EGFR mutation; MD, moderately differentiated; PD, poorly differentiated; TNM, tumour, node, metastasis (American Joint Committee on Cancer [AJCC]) stage; WD, well‐differentiated.

* *p* < 0.05 as calculated by competing‐risks regression.

Given the association between CD151 expression and advanced TNM stage in the NUH cohort, we next investigated survival differences according to early versus advanced stage disease in the high CD151, no EGFR mutations subgroup. For the early (TNM Stages I and II) stage patients, there was no significant difference between both subgroups (Figure 1D, *p* = 0.261). For the advanced (TNM Stages III and IV) state patients, the high CD151, no EGFR mutations subgroup had a much higher risk of death due to disease than the other subgroups (Sub‐HR [95% CI] 16.19 [3.71–70.63], *p* < 0.001; Figure 1D, Table 4).

**TABLE 4:** Competing‐risks regression of time to death due to disease for high CD151, no epidermal growth factor receptor (EGFR) mutations group in early or advanced tumour, node, metastasis (TNM, American Joint Committee on Cancer [AJCC]) stage versus others in the Singaporean cohort.

	Early TNM stage		Advanced TNM stage	
	Subhazard ratio (95% CI)	*p* Value	Subhazard ratio (95% CI)	*p* Value
Age	1.02 (0.97–1.08)	0.34	1.02 (0.98–1.07)	0.362
Sex				
Female	1	—	1	—
Male	0.80 (0.33–1.95)	0.626	0.72 (0.27–1.95)	0.52
Ethnicity				
Chinese	1	—	1	—
Others	0.23 (0.03–1.68)	0.146	0.10 (0.02–0.43)	**0.002** *
Smoking history				
Non‐smoker	1	—	1	—
Ex‐smoker	0.83 (0.16–4.37)	0.823	0.92 (0.19–4.49)	0.923
Smoker	0.99 (0.38–2.62)	0.988	0.79 (0.27–2.32)	0.667
pT stage				
T1, T2a	1	—	1	—
T2b, T3	0.37 (0.08–1.80)	0.219	0.99 (0.29–2.44)	0.989
Tumour grade				
WD	1	—	1	—
MD	1.04 (0.38–2.83)	0.94	0.62 (0.19–2.01)	0.423
PD	2.36 (0.55–10.13)	0.249	0.71 (0.14–3.52)	0.675
Subgroup				
Others	1	—	1	—
High CD151, no EGFR mut	1.72 (0.67–4.40)	0.261	16.19 (3.71–70.63)	**<0.001** *

*Note*: Reference factors are stated first. Bold indicates siginificant value (*p* < 0.05).

Abbreviations: CI, confidence interval; EGFR mut, EGFR mutation; MD, moderately differentiated; PD, poorly differentiated; pT, primary tumour; WD, well‐differentiated.

* *p* < 0.05 as calculated by competing‐risks regression.

To confirm our findings, we analysed another set of lung cancer patients from a public database called TCGA LUAD. These patients also did not have EGFR mutations. We found that patients with high CD151 levels had a higher rate of disease‐specific death from lung cancer than those with low CD151 levels (*p* = 0.045, Figure 1E), supporting our conclusion that CD151 overexpression is of prognostic value for lung cancer patients without EGFR mutations.

### 3.2 CD151 colocalizes and associates with EGFR

**FIGURE 2:**
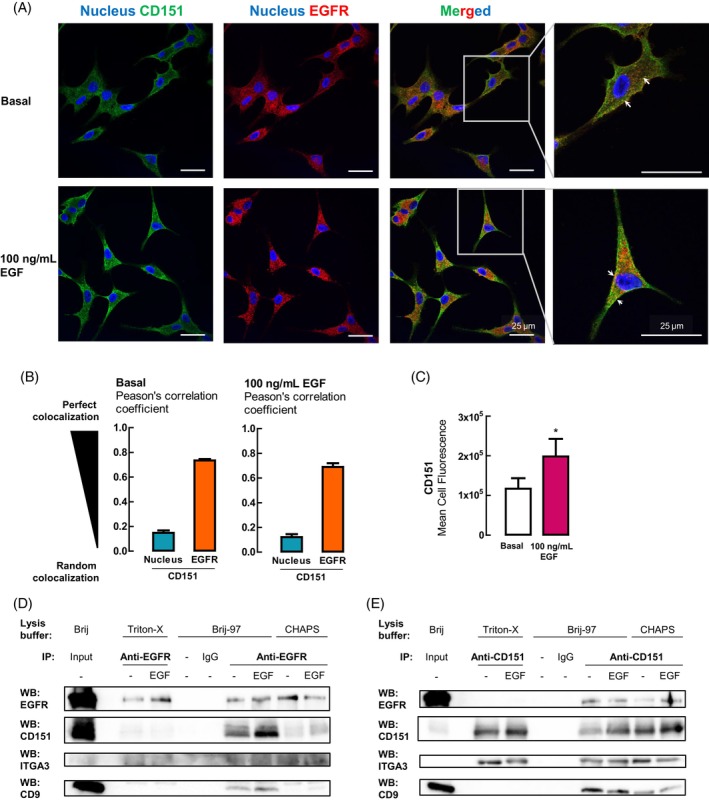
CD151 colocalizes and associates with epidermal growth factor receptor (EGFR). (A) Immunofluorescent images of A549 cells, which lack EGFR mutations, with nuclear (blue), CD151 (green) and EGFR (red) staining under basal and epidermal growth factor (EGF)‐stimulated conditions for 48 h. All scale bars represent 25 μm. (B) Colocalization analysis between CD151 and EGFR were determined by Pearson's correlation coefficient on ImageJ (*n* = 5 fields of view). (C) Mean cell fluorescence for CD151 was determined using ImageJ (*n* = 20 cells per treatment). *p < 0.05 as calculated by paired two‐tailed Student's *t*‐test. (D, E) Binding association of CD151 and EGFR respectively determined via immunoprecipitation (IP) and immunoblot for both proteins as well as known partner proteins of CD151 (integrin α3 and CD9) with or without treatment with 100 ng/mL EGF for 48 h.

We performed immunofluorescence to visualise the biological relationship between these CD151 and EGFR in A549 cells, which have no known EGFR mutations. Under both basal and EGF‐treated conditions, CD151 was found to colocalize with EGFR (Figure 2A,B). Furthermore, the mean cell fluorescence of CD151 in A549 cells was significantly increased with EGF treatment (Figure 2C).

To delve deeper into the nature of the association between CD151 and EGFR, we performed co‐immunoprecipitation (co‐IP) experiments. These revealed that CD151 associates with EGFR under Brij‐97 and CHAPS conditions but not under Triton‐X conditions (Figure 2D) when EGFR was pulled down. Similarly, integrin α3 was detected under the Brij‐97 and CHAPS conditions. In the reverse co‐IP experiments, where CD151 was pulled down, EGFR was also detected under the same detergent conditions (Brij‐97 and CHAPS, but not Triton‐X lysis) (Figure 2E). Interestingly, the binding association between CD151 and EGFR mirrors that of the tetraspanin‐tetraspanin association between CD151 and CD9, which was also maintained only in Brij‐97 and CHAPS (Figure 2E). As a positive control, we verified that CD151 co‐immunoprecipitates with integrin α3, which is a known strong binding partner of CD151, under all three detergent conditions^31^, ^32^ (Figure 2E). The EGFR‐integrin α3 association above has previously been reported in HeLa cells^33^ and could suggest that the CD151‐EGFR interaction exists within the tetraspanin‐enriched microdomain.^34^

Thus, this study underscores the potential significance of CD151 in the context of EGFR signalling in NSCLC.

### 3.3 CD151 is necessary and sufficient for EGF‐induced cell number

**FIGURE 3:**
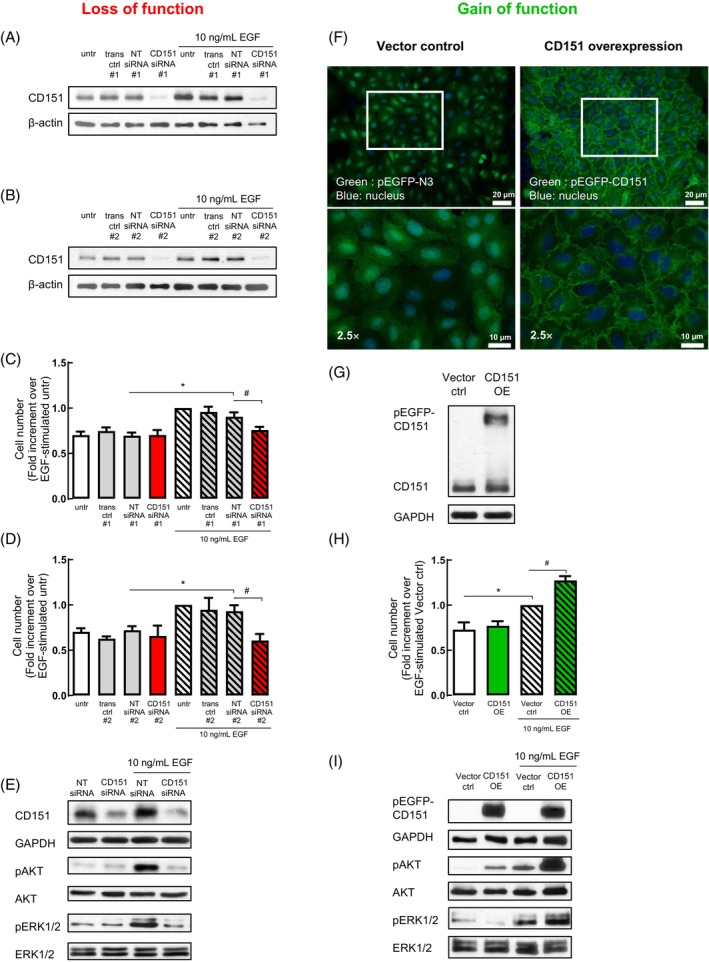
CD151 is required and sufficient for epidermal growth factor (EGF)‐induced cell number. Loss‐of‐function experiments were conducted in A549 cells that were transfected with commercially available CD151 siRNA. (A, B) Representative blots confirming CD151 protein knockdown using CD151 siRNA from (A) Dharmacon and (B) Qiagen 24 h post‐transfection. (C, D) Cells that were transfected with CD151 siRNA were then treated with 10 ng/mL EGF for 48 h to determine cell number changes under the different conditions. (E) Representative blots showing the effect of EGF stimulation for 5 min in cells transfected with CD151 siRNA 24 h prior. Gain‐of‐function experiments were conducted by generating stably CD151 overexpressing cells using vector plasmids. (F) Immunofluorescence images and (G) representative Western blot confirming CD151‐overexpressing (CD151 OE) in cells expressing GFP‐tagged CD151 (pEGFP‐CD151) compared with control cells (vector ctrl) overexpressing the vector (pEGFP‐N3). (H) Cell number was measured post‐EGF stimulation in both cell lines as well as (I) corresponding changes in signalling proteins via Western blot following EGF stimulation for 5 min. *,^#^ *p* < 0.05 as calculated by repeated measures one‐way ANOVA with Bonferroni's post hoc test on selected columns. Ctrl, control; NT, non‐targeting; trans, transfection; untr, untreated.

In view of the clinical significance of the high CD151, no EGFR mutation subtype, along with the relationship between CD151 and EGFR, we utilised NSCLC cells lacking an EGFR mutation to further explore this functional relationship. To do so, we performed loss‐of‐function studies whereby CD151 expression was knockdown with commercially available siRNAs in A549 cells (Figure 3A,B). This resulted in a significant ablation of EGF‐induced cell number (Figure 3C,D). Along with this, the levels of activated AKT (pAKT) and ERK1/2 (pERK1/2), two proteins involved in cell growth and survival, were also significantly reduced (Figure 3E).

In our gain‐of‐function studies, we created A549 cells that stably overexpress CD151. Overexpression of CD151 in the cells was verified using immunofluorescence (Figure 3F) and immunoblotting analysis (Figure 3G). CD151 OE cells were more sensitive to EGF stimulation compared to the vector control cells (Figure 3H) and showed increased levels of activated AKT and ERK1/2 (Figure 3I). These findings suggest that CD151 plays a crucial role in EGF‐induced cell proliferation, likely through its influence on the AKT and ERK1/2 signalling pathways. This highlights the potential of CD151 as a target for lung cancer treatment.

### 3.4 CD151 expression levels, through modulation or at baseline, influences the anti‐proliferative effects of erlotinib

**FIGURE 4:**
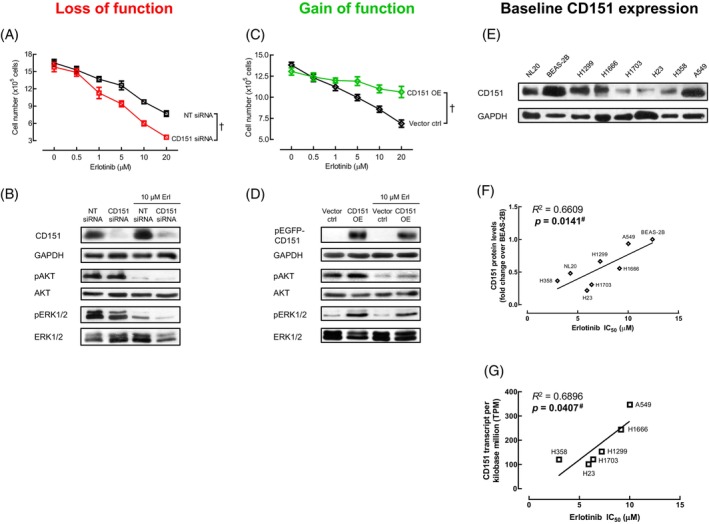
CD151 expression levels, through modulation or at baseline, influence erlotinib's anti‐proliferative effects. (A) Cell number following treatment with increasing erlotinib concentration for 72 h was measured in A549 cells transfected with non‐targeting (NT) or CD151 siRNA 24 h prior to erlotinib treatment. ^†^ *p* < 0.05 as calculated by two‐way ANOVA. (B) Representative blots showing the effect of combination CD151 siRNA transfection and erlotinib treatment in A549 cells for 5 min on protein expression. (C, D) Gain‐of‐function experiment was conducted with vector control (Vector ctrl) and CD151‐overexpressing (CD151 OE) cells to determine erlotinib response under these conditions in terms of (C) cell number and (D) protein expression. ^†^ *p* < 0.05 as calculated by two‐way ANOVA (*n* = 3–6) (E) Representative blot showing baseline CD151 protein levels for eight cell lines without EGFR mutations. (F) Baseline CD151 protein levels and (G) baseline CD151 RNA data (sourced from NIH Genomic Data Commons) were plotted against their respective erlotinib inhibitory concentrations (IC_50_). *R* ^2^ (Pearson's correlation coefficient) and *p*‐value was determined by linear regression analysis (^#^ *p* < 0.05). TNM, tumour, node, metastasis (American Joint Committee on Cancer [AJCC]) stage.

Given the key role of CD151 in EGF‐induced proliferation, we next assessed the effect of CD151 modulation on responsiveness to EGFR inhibition. Following treatment with EGFR‐specific TKI, erlotinib, we observed a greater reduction in cell number in the CD151 knockdown A549 cells compared to their non‐targeting control (Figure 4A). CD151 knockdown in the same A549 cells also reduced pAKT and pERK1/2 levels with erlotinib (Figure 4B). Enhancement of erlotinib cytotoxicity was observed in two other NSCLC cells that lack EGFR mutations, H358 and H1299 (Figure S2A,B).

The cytotoxic effect of erlotinib was found to be similar in both vector control cells and the parental cell line. However, cells overexpressing CD151 were less affected by the anti‐proliferative effects of erlotinib (Figure 4C). This is consistent with the results of CD151 knockdown experiments, where the reduction in the levels of activated AKT (pAKT) and ERK1/2 (pERK1/2) proteins, triggered by erlotinib, was less pronounced in cells overexpressing CD151 (Figure 4D). In addition to modulating CD151 levels, we also screened eight NSCLC cell lines that lacked EGFR mutations but had varying baseline CD151 levels (Figure 4E). We measured their respective half‐maximal inhibitory concentrations (IC_50_) for erlotinib (Figure S3A–H). We found a positive correlation between CD151 protein levels (normalised to BEAS‐2B) and erlotinib IC_50_ (Figure 4E,F). This correlation was confirmed using CD151 RNA from the National Institutes of Health (NIH) Genomic Data Commons database (Ref. 22360905 EBI UK) (Figure 4G). These findings underscore the importance of CD151 expression in determining the anti‐proliferative effect of erlotinib, especially in the absence of EGFR mutations. This suggests that CD151 could be a potential target for enhancing the effectiveness of erlotinib treatment in NSCLC, particularly when EGFR mutations are not detected.

## 4 DISCUSSION

Our study is the first to demonstrate a strong association between CD151 expression and the status of EGFR mutation, specifically noting a higher prevalence of CD151 expression in tumours lacking these mutations. We found significant associations between CD151 overexpression and factors such as smoking status, Chinese ethnicity and advanced TNM stage. The increased prevalence of high CD151 expression across East Asian cohorts, including Japanese,^26^ South Korean^25^ and Chinese^35^ populations, highlights its particular relevance in these demographics and underscores the need for further research to uncover contributing factors.

Consistent with previous studies,^25^, ^35^ our research reaffirms the prognostic significance of CD151 overexpression in NSCLC, emphasising its role in survival outcomes. Further stratification revealed that the relationship between high CD151 expression and the absence of EGFR mutations has clinical relevance, given the poor survival outcomes of this subgroup, particularly in patients with advanced TNM stages. This association may suggest a link between this tumour subtype and tumour aggressiveness. However, our study has limitations that need to be addressed in future research, including the uneven distribution of patients across stages (with 59% of patients at Stage I), the lack of information on treatment regimens, and the genetically diverse population of patients without EGFR mutations, which may include tumours driven by other oncogenes such as ALK, RAS and ROS1.^3^ Despite these limitations, our findings underscore the potential of CD151 overexpression as a prognostic tool, particularly for stratifying the subgroup of patients without EGFR mutations. This could have significant implications for disease prognosis and treatment strategies in NSCLC.

The association between CD151 and EGFR has been initially examined through immunohistochemistry in patient samples of oral squamous cell carcinoma^36^ and invasive breast cancer.^37^ Additionally, the co‐expression of CD151 and p‐EGFR in NSCLC has been reported,^35^ aligning with our current findings, where we observed an increase in CD151 expression with EGFR activation. We show for the first time, a colocalization of CD151 and EGFR, specifically in NSCLC cells without EGFR mutations. CD151 is known to exert its diverse downstream effects via adhesion to various binding partners.^38^ These interactions between tetraspanins and partner proteins can be categorised into three major types: first‐, second‐ and third‐level interactions, such as CD151‐integrin α3β1 complex,^39^ tetraspanin‐tetraspanin complex^40^ and CD151‐PI4K,^41^ respectively. Whilst one group has reported on the binding association between CD151 and p‐EGFR,^35^ our study distinctively employed a comprehensive co‐IP strategy with three different detergents to elucidate the strength of this association. This approach has allowed us to infer that the strength and type of interaction between CD151 and EGFR may mirror that of tetraspanin‐tetraspanin interactions, as evidenced by the similarities in conditions for CD151‐EGFR and CD151‐CD9 co‐IP results.

Several studies in breast cancer, skin squamous cell carcinoma and glioblastoma have delved into the functional relationship between CD151 and EGFR^18^, ^42^, ^43^ in mediating cell spreading, invasion, colony formation or tumorigenesis. This knowledge has been extended to NSCLC, where it was shown that CD151 is involved in regulating EGFR/ErbB2 signalling pathways, influencing NSCLC cell proliferation, migration and invasion.^35^ This corroborates the findings from our current study on the role of CD151 in EGF‐mediated proliferation in an NSCLC model without EGFR mutations. Other groups have reported CD151's involvement in HGF‐induced A549 cell proliferation by regulating ERK1/2,^44^ whilst the role of CD151 in the activation of AKT and ERK1/2 has varied, with some studies showing the requirement of one but not the other, or both.^45^, ^46^, ^47^ Our study demonstrated that CD151 is both necessary and sufficient for EGF‐induced cell proliferation through AKT and ERK1/2 activation.

Our study demonstrated that variations in CD151 expression, whether through modulation or baseline levels, influence the anti‐proliferative effects of the EGFR, erlotinib. This is likely due to a dysregulated interaction between CD151 and EGFR. This finding supports our clinical observations of a robust association between CD151 expression and EGFR status. Whilst erlotinib is widely recognised as the first‐line therapy for advanced NSCLC patients with positive EGFR mutations, it has also shown clinical benefits in other settings, such as maintenance therapy and as a second or third‐line treatment.^48^, ^49^, ^50^ However, no validated predictive biomarker exists for erlotinib response in patients lacking EGFR mutations. Our study proposes overexpression of CD151 as a novel predictive biomarker for erlotinib activity. We found that inhibiting CD151 sensitises NSCLC cells lacking EGFR mutations to erlotinib, further emphasising the significance of CD151 in this therapeutic context. This is consistent with findings that CD151 knockdown can enhance the sensitivity of NSCLC cells to inhibitors like gefitinib, suggesting a potential role for CD151 in improving clinical outcomes in NSCLC patients.^35^ Other studies have identified factors, such as cisplatin pre‐treatment^51^ and the compound shikonin,^52^ that sensitise NSCLC cells lacking EGFR mutations to TKI therapy. Therefore, our study represents another promising avenue for developing NSCLC therapeutics, especially considering the well‐characterised safety and toxicity profiles of these already available therapeutics.

Given the clinical significance of CD151 in several disease pathophysiologies, recent preclinical studies have reported the potential of targeted CD151 therapy (these overall findings have been reviewed previously^19^, ^53^, ^54^). In CD151‐null mice, both experimental and spontaneous lung metastasis were reduced,^18^, ^46^ whilst CD151‐targeted monoclonal antibodies have also shown a reduction in metastasis,^54^ delayed tumour progression^45^ and decreased tumorigenesis.^55^ Our study adds another piece to the complex puzzle of the underlying mechanism by which CD151 contributes to NSCLC and brings this field one step closer to the clinical stage. Notably, CD151 may be a promising systemic therapeutic strategy that may be useful in metastatic disease, given that targeting this protein has widespread anticancer activity in organs beyond the lungs. However, the widespread distribution of CD151 is a double‐edged sword, as considerations for targeted drug delivery must be taken to prevent unwanted, adverse effects to other healthy cells or organs.

## 5 CONCLUSIONS

Our study reveals a notable link between the overexpression of CD151 and the lack of EGFR mutations in NSCLC patients. This specific type of tumour, characterised by high CD151 and the absence of EGFR mutations, was associated with poorer survival outcomes in patients from both our study cohorts. On a functional level, we demonstrated that CD151 and EGFR colocalize and interact to regulate cell number induced by EGF. This regulation is dependent on the activation of AkT and ERK1/2, two proteins implicated in cell growth and survival. Importantly, our findings highlight the role of CD151 expression in determining the effectiveness of TKIs, particularly in cells that lack EGFR mutations. In summary, our research sheds light on the clinical and functional importance of the complex relationship between high CD151 expression and the absence of EGFR mutations. This intersection suggests that CD151 overexpression could serve as a robust prognostic biomarker, paving the way for its potential as a novel therapeutic target and management strategy, particularly relevant in NSCLC patients without EGFR mutations.

## AUTHOR CONTRIBUTIONS

Conceptualization: TT and AH‐PW; methodology: AH‐PW, MEN, CYC, YKT; software: OA, HY; validation: AH‐PW, MEN, CYC, YKT; formal analysis: AH‐PW, MEN, WHC, OA, HY; JKCT; investigation: AH‐PW, MEN, CYC, YKT; resources: JKCT, TT; data curation: RS, OA, HY, JKCT, JES, YCL, TT; writing—original draft preparation: AH‐PW and TT; writing—review and editing: AH‐PW, MEN, CYC, YKT, HCW, RS, OA, HY, JES, YCL, JKCT, TT; visualisation: AH‐PW, MEN, CYC, YKT; supervision: TT; project administration: JKCT, TT; funding acquisition: JKCT, TT. All authors have read and agreed to the published version of the manuscript.

## CONFLICT OF INTEREST STATEMENT

The authors declare no conflict of interest.

## Supporting information


**Figure S1.** Inclusion criteria for NSCLC adenocarcinoma patients in the Singaporean cohort. Patients were recruited from 1989 to 2011 (median year 2008, mean year 2005) in the National University Hospital (NUH), Singapore. *157 cases were included in statistical analysis. NSCLC, non‐small cell lung cancer; IHC, immunohistochemical analysis; EGFR, epidermal growth factor receptor.

**Figure S2.** Enhancement of erlotinib cytotoxicity in NSCLC cells that lack EGFR mutations. (A, B) Loss‐of‐function experimental set up with erlotinib was repeated by measuring effect of combination siRNA transfection and erlotinib treatment on cell number in two NSCLC cells that lack EGFR mutations, H358 and H1299, with corresponding blots to confirm CD151 knockdown (*n* = 3–4). Corresponding blots to confirm CD151 protein reduction with siRNA are shown. ^†^ *p* < 0.05 as calculated by two‐way Analysis of variance; GFP = green fluorescent protein (ANOVA).

**Figure S3.** Basal CD151 expression of NSCLC cells that lack EGFR mutation is associated with erlotinib efficacy. (A–H) Various NSCLC cells that lack EGFR mutations were treated with increasing concentrations of erlotinib for 72 h before cell enumeration via trypan blue exclusion. These cell lines include two normal cell lines, (A) BEAS‐2B and (B) NL‐20, and six NSCLC cell lines, (C) H1299, (D) H1666, (E) H1703, (F) H23, (G) H358 and (H) A549. Data are expressed as raw cell number and log transformed to determine IC_50_ of erlotinib (that is, the concentration of drug required to reduce cell number by 50%). *p < 0.05 as calculated by repeated measures one‐way ANOVA with Dunnett's post hoc test compared with control (*n* = 3–5).

**Table S1.** Patient demographics for Singaporean cohort.

**Table S2.** Patient demographics for TCGA cohort.

**Data S1.** The following supporting information can be downloaded at [link]: Supplementary Methods (Measurement for mean cell fluorescence and colocalization; Cell lines and reagents; Gene silencing by small‐interfering RNA; Generation of pEGFP‐CD151(CD151‐overexpressing) cell lines).

## Data Availability

The data that support the findings of this study are available from the corresponding author upon reasonable request.

## References

[1] Walters S , Maringe C , Coleman MP , et al. Lung cancer survival and stage at diagnosis in Australia, Canada, Denmark, Norway, Sweden and the UK: a population‐based study, 2004‐2007. Thorax. 2013;68(6):551‐564. doi:10.1136/thoraxjnl-2012-202297 23399908

[2] Sung H , Ferlay J , Siegel RL , et al. Global cancer statistics 2020: GLOBOCAN estimates of incidence and mortality worldwide for 36 cancers in 185 countries. CA Cancer J Clin. 2021;71(3):209‐249. doi:10.3322/caac.21660 33538338

[3] Travis WD , Rekhtman N . Pathological diagnosis and classification of lung cancer in small biopsies and cytology: strategic management of tissue for molecular testing. Semin Respir Crit Care Med. 2011;32(1):22‐31. doi:10.1055/s-0031-1272866 21500121

[4] Jacobsen MM , Silverstein SC , Quinn M , et al. Timeliness of access to lung cancer diagnosis and treatment: a scoping literature review. Lung Cancer. 2017;112:156‐164. doi:10.1016/j.lungcan.2017.08.011 29191588

[5] Alexander M , Kim SY , Cheng H . Update 2020: management of non‐small cell lung cancer. Lung. 2020;198(6):897‐907. doi:10.1007/s00408-020-00407-5 33175991 PMC7656891

[6] Arbour KC , Riely GJ . Systemic therapy for locally advanced and metastatic non‐small cell lung cancer: a review. Jama. 2019;322(8):764‐774. doi:10.1001/jama.2019.11058 31454018

[7] Kang Y , Jin Y , Li Q , Yuan X . Advances in lung cancer driver genes associated with brain metastasis. Front Oncol. 2020;10:606300. doi:10.3389/fonc.2020.606300 33537237 PMC7848146

[8] Ettinger DS , Wood DE , Aisner DL , et al. NCCN guidelines insights: non‐small cell lung cancer, version 2.2021. J Natl Compr Cancer Netw. 2021;19(3):254‐266. doi:10.6004/jnccn.2021.0013 33668021

[9] Herbst RS , Bunn PA . Targeting the epidermal growth factor receptor in non‐small cell lung cancer. Clin Cancer Res. 2003;9(16 pt 1):5813‐5824. 14676101

[10] Rosell R , Carcereny E , Gervais R , et al. Erlotinib versus standard chemotherapy as first‐line treatment for European patients with advanced EGFR mutation‐positive non‐small‐cell lung cancer (EURTAC): a multicentre, open‐label, randomised phase 3 trial. Lancet Oncol. 2012;13(3):239‐246. doi:10.1016/S1470-2045(11)70393-X 22285168

[11] Mok TS , Wu YL , Thongprasert S , et al. Gefitinib or carboplatin‐paclitaxel in pulmonary adenocarcinoma. N Engl J Med. 2009;361(10):947‐957. doi:10.1056/NEJMoa0810699 19692680

[12] Graham RP , Treece AL , Lindeman NI , et al. Worldwide frequency of commonly detected EGFR mutations. Arch Pathol Lab Med. 2018;142(2):163‐167. doi:10.5858/arpa.2016-0579-CP 29106293

[13] Werutsky G , Debiasi M , Sampaio FH , et al. P1.08: updated analysis of global epidemiology of EGFR mutation in advanced non‐small cell lung cancer: track: prevention, early detection, epidemiology and tobacco control. J Thorac Oncol. 2016;11(10):S184‐S185. doi:10.1016/j.jtho.2016.08.030

[14] Zhang T , Wan B , Zhao Y , et al. Treatment of uncommon EGFR mutations in non‐small cell lung cancer: new evidence and treatment. Transl Lung Cancer Res. 2019;8(3):302‐316. 31367543 10.21037/tlcr.2019.04.12 PMC6626855

[15] Tomasini P , Brosseau S , Mazières J , et al. EGFR tyrosine kinase inhibitors *versus* chemotherapy in *EGFR* wild‐type pre‐treated advanced nonsmall cell lung cancer in daily practice. Eur Respir J. 2017;50(2):1700514. doi:10.1183/13993003.00514-2017 28798090

[16] Bar J , Peled N , Schokrpur S , et al. UNcommon EGFR mutations: International case series on efficacy of Osimertinib in Real‐life practice in first‐LiNe setting (UNICORN). J Thorac Oncol. 2023;18(2):169‐180. doi:10.1016/j.jtho.2022.10.004 36307041

[17] Zhou F , Zhou C‐C . Targeted therapies for patients with advanced NSCLC harboring wild‐type EGFR: what's new and what's enough. Chin J Cancer. 2015;34(3):31‐319. doi:10.1186/s40880-015-0036-4 26187152 PMC4593374

[18] Yang XH , Richardson AL , Torres‐Arzayus MI , et al. CD151 accelerates breast cancer by regulating α(6) integrin function, signaling, and molecular organization. Cancer Res. 2008;68(9):3204‐3213. doi:10.1158/0008-5472.CAN-07-2949 18451146 PMC4764302

[19] Wong AH , Tran T . CD151 in respiratory diseases. Front Cell Dev Biol. 2020;8:64. doi:10.3389/fcell.2020.00064 32117989 PMC7020194

[20] Hemler ME . Tetraspanin proteins promote multiple cancer stages. Nat Rev Cancer. 2014;14(1):49‐60. doi:10.1038/nrc3640 24505619

[21] Sadej R , Grudowska A , Turczyk L , Kordek R , Romanska HM . CD151 in cancer progression and metastasis: a complex scenario. Lab Invest. 2014;94(1):41‐51. doi:10.1038/labinvest.2013.136 24247563

[22] Ang J , Lijovic M , Ashman LK , Kan K , Frauman AG . CD151 protein expression predicts the clinical outcome of low‐grade primary prostate cancer better than histologic grading: a new prognostic indicator? Cancer Epidemiol Biomarkers Prev. 2004;13(11 pt 1):1717‐1721. 15533898

[23] Ke AW , Shi GM , Zhou J , et al. Role of overexpression of CD151 and/or c‐Met in predicting prognosis of hepatocellular carcinoma. Hepatology. 2009;49(2):491‐503. doi:10.1002/hep.22639 19065669

[24] Sincock PM , Mayrhofer G , Ashman LK . Localization of the transmembrane 4 superfamily (TM4SF) member PETA‐3 (CD151) in normal human tissues: comparison with CD9, CD63, and alpha5beta1 integrin. J Histochem Cytochem. 1997;45(4):515‐525. doi:10.1177/002215549704500404 9111230

[25] Kwon MJ , Seo J , Kim YJ , et al. Prognostic significance of CD151 overexpression in non‐small cell lung cancer. Lung Cancer. 2013;81(1):109‐116. doi:10.1016/j.lungcan.2013.03.014 23570797

[26] Tokuhara T , Hasegawa H , Hattori N , et al. Clinical significance of *CD151* gene expression in non‐small cell lung cancer. Clin Cancer Res. 2001;7(12):4109‐4114. 11751509

[27] Das K , Omar MFM , Ong CW , et al. TRARESA: a tissue microarray‐based hospital system for biomarker validation and discovery. Pathology. 2008;40(5):441‐449. doi:10.1080/00313020802198101 18604728

[28] Lee D , Suh YL , Park TI , et al. Prognostic significance of tetraspanin CD151 in newly diagnosed glioblastomas. J Surg Oncol. 2012;107(6):646‐652. doi:10.1002/jso.23249 22926763

[29] Omar MF , Ito K , Nga ME , et al. RUNX3 downregulation in human lung adenocarcinoma is independent of p53, EGFR or KRAS status. Pathol Oncol Res. 2012;18(4):783‐792. doi:10.1007/s12253-011-9485-5 22729835

[30] Tran T , Teoh CM , Tam JKC , et al. Laminin drives survival signals to promote a contractile smooth muscle phenotype and airway hyperreactivity. FASEB J. 2013;27(10):3991‐4003. doi:10.1096/fj.12-221341 23756649 PMC6159668

[31] Tran T , Stewart AG . Protease‐activated receptor (PAR)‐independent growth and pro‐inflammatory actions of thrombin on human cultured airway smooth muscle. Br J Pharmacol. 2003;138(5):865‐875. doi:10.1038/sj.bjp.0705106 12642388 PMC1573717

[32] Qiao Y , Tam JKC , Tan SSL , et al. CD151, a laminin receptor showing increased expression in asthmatic patients, contributes to airway hyperresponsiveness through calcium signaling. J Allergy Clin Immunol. 2017;139(1):82‐92.e5. doi:10.1016/j.jaci.2016.03.029 27233153

[33] Hang Q , Isaji T , Hou S , Zhou Y , Fukuda T , Gu J . N‐Glycosylation of integrin α5 acts as a switch for EGFR‐mediated complex formation of integrin α5β1 to α6β4. Sci Rep. 2016;6(1):33507. doi:10.1038/srep33507 27641064 PMC5027594

[34] Termini CM , Gillette JM . Tetraspanins function as regulators of cellular signaling. Front Cell Dev Biol. 2017;5:34. doi:10.3389/fcell.2017.00034 28428953 PMC5382171

[35] Zhu J , Cai T , Zhou J , et al. CD151 drives cancer progression depending on integrin α3β1 through EGFR signaling in non‐small cell lung cancer. J Exp Clin Cancer Res. 2021;40(1):192. doi:10.1186/s13046-021-01998-4 34108040 PMC8191020

[36] Romanska HM , Potemski P , Collins SI , Williams H , Parmar S , Berditchevski F . Loss of CD151/Tspan24 from the complex with integrin alpha3beta1 in invasive front of the tumour is a negative predictor of disease‐free survival in oral squamous cell carcinoma. Oral Oncol. 2013;49(3):224‐229. doi:10.1016/j.oraloncology.2012.09.013 23099281

[37] Kwon MJ , Park S , Choi JY , et al. Clinical significance of CD151 overexpression in subtypes of invasive breast cancer. Br J Cancer. 2012;106(5):923‐930. doi:10.1038/bjc.2012.11 22294188 PMC3306846

[38] te Molder L , Juksar J , Harkes R , Wang W , Kreft M , Sonnenberg A . Tetraspanin CD151 and integrin α3β1 contribute to the stabilization of integrin α6β4‐containing cell‐matrix adhesions. J Cell Sci. 2019;132(19):1‐15. doi:10.1242/jcs.235366 31488507

[39] Yauch RL , Kazarov AR , Desai B , Lee RT , Hemler ME . Direct extracellular contact between integrin alpha(3)beta(1) and TM4SF protein CD151. J Biol Chem. 2000;275(13):9230‐9238. doi:10.1074/jbc.275.13.9230 10734060

[40] Yanez‐Mo M , Alfranca A , Cabañas C , et al. Regulation of endothelial cell motility by complexes of tetraspan molecules CD81/TAPA‐1 and CD151/PETA‐3 with alpha3 beta1 integrin localized at endothelial lateral junctions. J Cell Biol. 1998;141(3):791‐804. doi:10.1083/jcb.141.3.791 9566977 PMC2132738

[41] Yauch RL , Hemler ME . Specific interactions among transmembrane 4 superfamily (TM4SF) proteins and phosphoinositide 4‐kinase. Biochem J. 2000;351(pt 3):629‐637. 11042117 PMC1221402

[42] Li Q , Yang XH , Xu F , et al. Tetraspanin CD151 plays a key role in skin squamous cell carcinoma. Oncogene. 2013;32(14):1772‐1783. doi:10.1038/onc.2012.205 22824799 PMC3482293

[43] Zhou P , Erfani S , Liu Z , et al. CD151‐alpha3beta1 integrin complexes are prognostic markers of glioblastoma and cooperate with EGFR to drive tumor cell motility and invasion. Oncotarget. 2015;6(30):29675‐29693. doi:10.18632/oncotarget.4896 26377974 PMC4745755

[44] Franco M , Muratori C , Corso S , et al. The tetraspanin CD151 is required for Met‐dependent signaling and tumor cell growth. J Biol Chem. 2010;285(50):38756‐38764. doi:10.1074/jbc.M110.145417 20937830 PMC2998140

[45] Sadej R , Romanska H , Baldwin G , et al. CD151 regulates tumorigenesis by modulating the communication between tumor cells and endothelium. Mol Cancer Res. 2009;7(6):787‐798. doi:10.1158/1541-7786.MCR-08-0574 19531562

[46] Deng X , Li Q , Hoff J , et al. Integrin‐associated CD151 drives ErbB2‐evoked mammary tumor onset and metastasis. Neoplasia. 2012;14(8):678‐689. 22952421 10.1593/neo.12922 PMC3431176

[47] Takeda Y , Kazarov AR , Butterfield CE , et al. Deletion of tetraspanin Cd151 results in decreased pathologic angiogenesis in vivo and in vitro. Blood. 2007;109(4):1524. doi:10.1182/blood-2006-08-041970 17023588 PMC1794066

[48] Jazieh AR , Al Sudairy R , Abu‐Shraie N , Al Suwairi W , Ferwana M , Murad MH . Erlotinib in wild type epidermal growth factor receptor non‐small cell lung cancer: a systematic review. Ann Thorac Med. 2013;8(4):204‐208. doi:10.4103/1817-1737.118503 24250733 PMC3821279

[49] Shepherd FA , Rodrigues Pereira J , Ciuleanu T , et al. Erlotinib in previously treated non‐small‐cell lung cancer. N Engl J Med. 2005;353(2):123‐132. doi:10.1056/NEJMoa050753 16014882

[50] Reck M , van Zandwijk N , Gridelli C , et al. Erlotinib in advanced non‐small cell lung cancer: efficacy and safety findings of the global phase IV tarceva lung cancer survival treatment study. J Thorac Oncol. 2010;5(10):1616‐1622. doi:10.1097/JTO.0b013e3181f1c7b0 20736854

[51] Raimbourg J , Joalland MP , Cabart M , et al. Sensitization of EGFR wild‐type non‐small cell lung cancer cells to EGFR‐tyrosine kinase inhibitor erlotinib. Mol Cancer Ther. 2017;16(8):1634‐1644. doi:10.1158/1535-7163.Mct-17-0075 28522592

[52] Li YL , Hu X , Li QY , et al. Shikonin sensitizes wild‐type EGFR NSCLC cells to erlotinib and gefitinib therapy. Mol Med Rep. 2018;18(4):3882‐3890. doi:10.3892/mmr.2018.9347 30106133 PMC6131653

[53] Kumari S , Gayathri Devi V , Badana A , Dasari VR , Malla RR . CD151‐A striking marker for cancer therapy. Biomark Cancer. 2015;7:7‐11. doi:10.4137/bic.s21847 25861224 PMC4372031

[54] Haeuw JF , Goetsch L , Bailly C , Corvaia N . Tetraspanin CD151 as a target for antibody‐based cancer immunotherapy. Biochem Soc Trans. 2011;39(2):553‐558. doi:10.1042/bst0390553 21428938

[55] Kohno M , Hasegawa H , Miyake M , Yamamoto T , Fujita S . CD151 enhances cell motility and metastasis of cancer cells in the presence of focal adhesion kinase. Int J Cancer. 2002;97(3):336‐343. 11774285 10.1002/ijc.1605

