# Supplementary material for: Impact of CD151 overexpression on prognosis and therapy in non‐small cell lung cancer patients lacking EGFR mutations
Source: Cell Prolif. 2024 Jul 9;57(9):e13708. doi: 10.1111/cpr.13708 (PMC11503249; doi:10.1111/cpr.13708)
Supplement: Supplementary file 1 — Figure S1. Inclusion criteria for NSCLC adenocarcinoma patients in the Singaporean cohort. Patients were recruited from 1989 to 2011 (median year 2008, mean year 2005) in the National University Hospital (NUH), Singapore. *157 cases were included in statistical analysis. NSCLC, non‐small cell lung cancer; IHC, immunohistochemical analysis; EGFR, epidermal growth factor receptor. [file CPR-57-e13708-s005.pptx]

## Slide 1
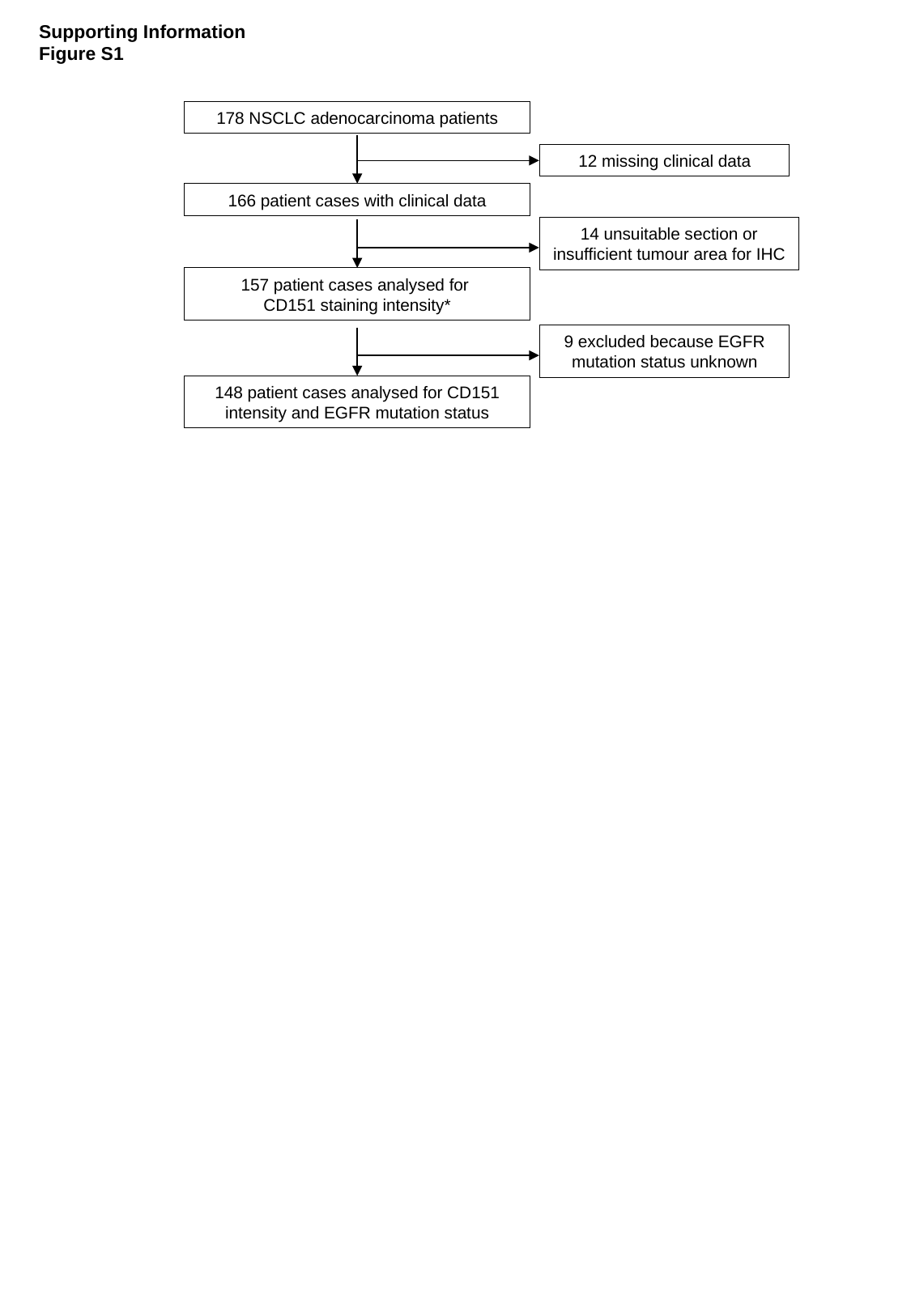

Supporting Information
Figure S1
178 NSCLC adenocarcinoma patients
12 missing clinical data
166 patient cases with clinical data
14 unsuitable section or insufficient tumour area for IHC
157 patient cases analysed for
CD151 staining intensity*
9 excluded because EGFR mutation status unknown
148 patient cases analysed for CD151 intensity and EGFR mutation status
