# Supplementary figures and images for: Impact of CD151 overexpression on prognosis and therapy in non‐small cell lung cancer patients lacking EGFR mutations
Source: Cell Prolif. 2024 Jul 9;57(9):e13708. doi: 10.1111/cpr.13708 (PMC11503249; doi:10.1111/cpr.13708)

## Slide 1
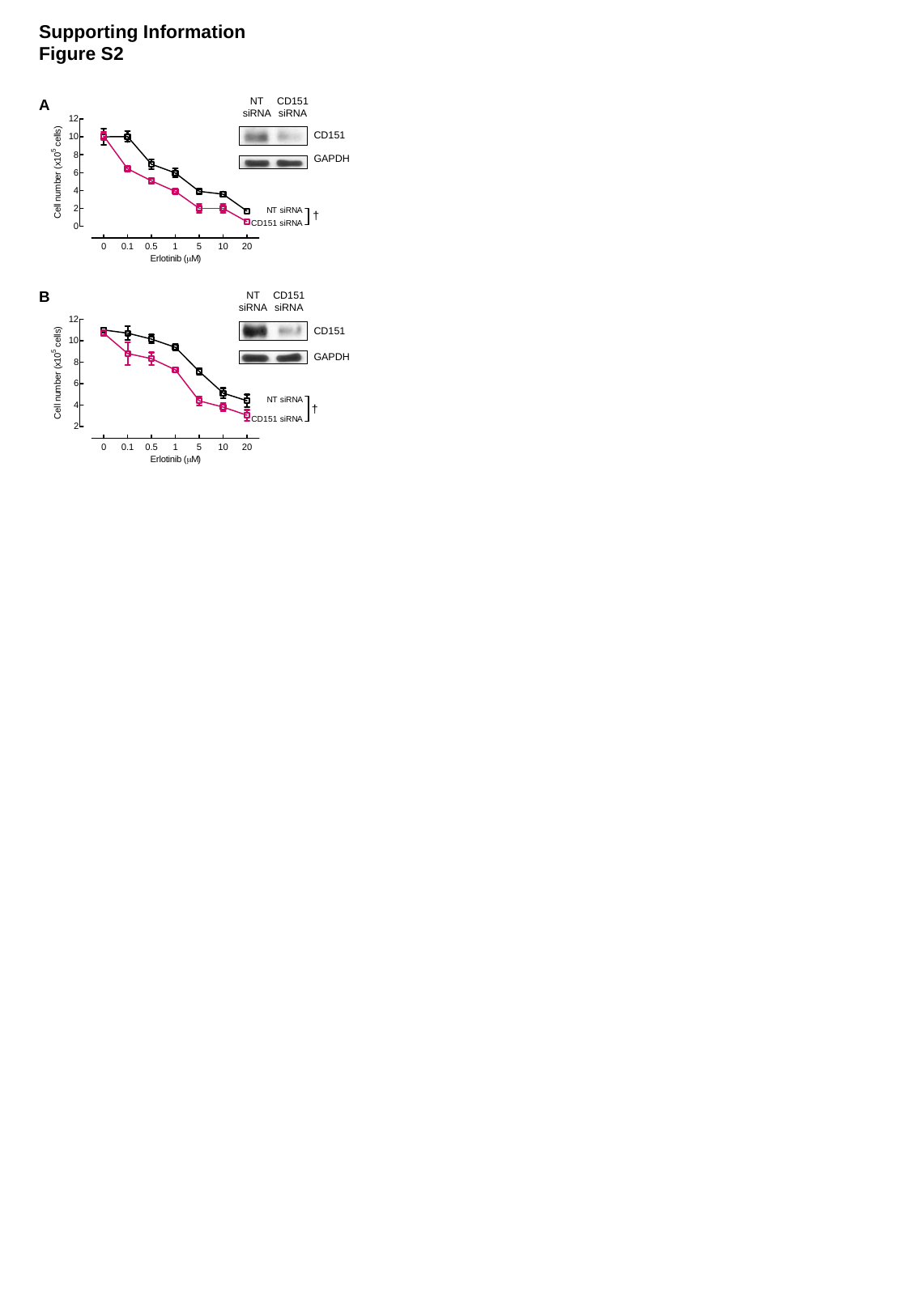

Supporting Information
Figure S2
NT
siRNA
CD151
siRNA
A
CD151
GAPDH
B
NT
siRNA
CD151
siRNA
CD151
GAPDH

Supplement: Supplementary file 2 — Figure S2. Enhancement of erlotinib cytotoxicity in NSCLC cells that lack EGFR mutations. (A, B) Loss‐of‐function experimental set up with erlotinib was repeated by measuring effect of combination siRNA transfection and erlotinib treatment on cell number in two NSCLC cells that lack EGFR mutations, H358 and H1299, with corresponding blots to confirm CD151 knockdown (n = 3–4). Corresponding blots to confirm CD151 protein reduction with siRNA are shown. † p < 0.05 as calculated by two‐way Analysis of variance; GFP = green fluorescent protein (ANOVA). [file CPR-57-e13708-s001.pptx]

## Slide 1
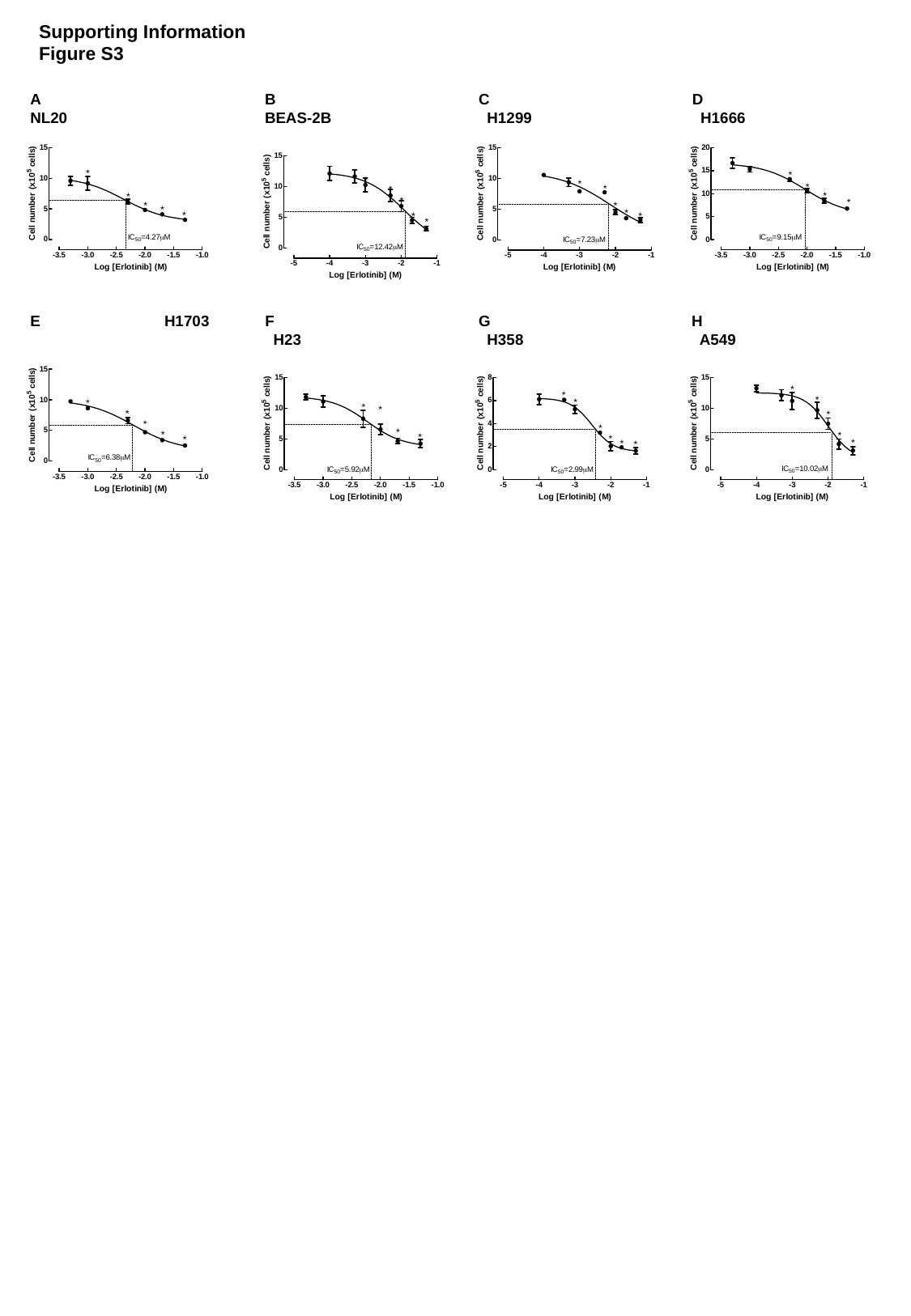

Supporting Information
Figure S3
A	 NL20
B	 BEAS-2B
C	 H1299
D	 H1666
E	 H1703
F	 H23
G	 H358
H	 A549

Supplement: Supplementary file 3 — Figure S3. Basal CD151 expression of NSCLC cells that lack EGFR mutation is associated with erlotinib efficacy. (A–H) Various NSCLC cells that lack EGFR mutations were treated with increasing concentrations of erlotinib for 72 h before cell enumeration via trypan blue exclusion. These cell lines include two normal cell lines, (A) BEAS‐2B and (B) NL‐20, and six NSCLC cell lines, (C) H1299, (D) H1666, (E) H1703, (F) H23, (G) H358 and (H) A549. Data are expressed as raw cell number and log transformed to determine IC50 of erlotinib (that is, the concentration of drug required to reduce cell number by 50%). *p < 0.05 as calculated by repeated measures one‐way ANOVA with Dunnett's post hoc test compared with control (n = 3–5). [file CPR-57-e13708-s002.pptx]
