# Supplementary material for: Impact of CD151 overexpression on prognosis and therapy in non‐small cell lung cancer patients lacking EGFR mutations
Source: Cell Prolif. 2024 Jul 9;57(9):e13708. doi: 10.1111/cpr.13708 (PMC11503249; doi:10.1111/cpr.13708)
Supplement: Supplementary file 4 — Table S1. Patient demographics for Singaporean cohort. [file CPR-57-e13708-s003.docx]

**Table S1**

Patient demographics for Singaporean cohort

|  |  | **n=157** |
| --- | --- | --- |
| Age |  |  |
|  | Median (Range) | 63 (35-85) |
| Sex |  |  |
|  | Male | 94 (62%) |
|  | Female | 58 (38%) |
| Ethnicity | |  |
|  | Chinese | 114 (75%) |
|  | Others | 37 (25%) |
| Smoking history | |  |
|  | Non-smoker | 60 (51%) |
|  | Ex-smoker | 23 (19%) |
|  | Smoker | 35 (30%) |
| TNM stage | |  |
|  | I | 87 (60%) |
|  | II | 22 (15%) |
|  | III | 20 (14%) |
|  | IV | 16 (11%) |
| pT stage | |  |
|  | T1 | 64 (46%) |
|  | T2a | 57 (41%) |
|  | T2b | 8 (6%) |
|  | T3 | 9 (7%) |
| Tumour grade | |  |
|  | WD | 11 (8%) |
|  | MD | 97 (72%) |
|  | PD | 26 (19%) |
| EGFR subtype | |  |
|  | No EGFR mut | 79 (53%) |
|  | EGFR mut | 69 (47%) |
| CD151 expression | |  |
|  | Low | 53 (34%) |
|  | High | 102 (66%) |
|  |  |  |
|  |  |  |

Data are number (%) unless otherwise stated.

TNM, tumour, node, metastasis (AJCC) stage; pT, primary tumour; WD, well-differentiated, MD, moderately differentiated, PD, poorly differentiated; EGFR mut, EGFR mutation; EGFR, epidermal growth factor receptor.
