# Supplementary material for: Impact of CD151 overexpression on prognosis and therapy in non‐small cell lung cancer patients lacking EGFR mutations
Source: Cell Prolif. 2024 Jul 9;57(9):e13708. doi: 10.1111/cpr.13708 (PMC11503249; doi:10.1111/cpr.13708)
Supplement: Supplementary file 5 — Table S2. Patient demographics for TCGA cohort. [file CPR-57-e13708-s004.docx]

**Table S2**

Patient demographics for TCGA cohort

|  |  | **n=199** |
| --- | --- | --- |
| Sex |  |  |
|  | Male | 90 (45%) |
|  | Female | 109 (55%) |
| Ethnicity | |  |
|  | Caucasian | 191 (95%) |
|  | Others | 8 (4%) |
| Smoking history | |  |
|  | Non-smoker | 22 (11%) |
|  | Ex-smoker | 116 (61%) |
|  | Smoker | 53 (28%) |
| TNM stage | |  |
|  | I | 118 (59%) |
|  | II | 45 (23%) |
|  | III | 28 (14%) |
|  | IV | 8 (4%) |
| EGFR mutation status | |  |
|  | No EGFR mut | all |
| Tumour subtype | |  |
|  | Adenocarcinoma | all |
|  |  |  |
|  |  |  |

Data are number (%) unless otherwise stated.

TNM, tumour, node, metastasis (AJCC) stage; EGFR mut, EGFR mutation; EGFR, epidermal growth factor receptor.
