# Supplementary material for: Impact of CD151 overexpression on prognosis and therapy in non‐small cell lung cancer patients lacking EGFR mutations
Source: Cell Prolif. 2024 Jul 9;57(9):e13708. doi: 10.1111/cpr.13708 (PMC11503249; doi:10.1111/cpr.13708)
Supplement: Supplementary file 6 — Data S1. The following supporting information can be downloaded at [link]: Supplementary Methods (Measurement for mean cell fluorescence and colocalization; Cell lines and reagents; Gene silencing by small‐interfering RNA; Generation of pEGFP‐CD151(CD151‐overexpressing) cell lines). [file CPR-57-e13708-s006.docx]

Supplementary Methods

*Measurement for mean cell fluorescence and colocalization*

The corrected total cell fluorescence was calculated by subtracting the product of the area of the selected cell and mean fluorescence of background reading from the integrated density of the region of interest. The corrected total cell fluorescence for 20 cells per treatment condition was used to derive the mean cell fluorescence. Colocalization was measured as a function of Pearson’s correlation coefficient analysis, which was determined by splitting the channels on ImageJ then using the Just Another Colocalization Plugin (JACoP, NIH, USA). A coefficient of ‘0’ describes random colocalization whereas that of ‘1’ describes perfect colocalization.

*Cell lines and reagents*

Eight NSCLC cell lines that lack EGFR mutations (BEAS-2B, NL-20, A549, H23, H358, H1299, H1666, and H1703) from American Type Culture Collection (ATCC) were cultured in DMEM or RPMI-1640 (Gibco) supplemented with 10% v/v FBS and 1% penicillin-streptomycin. Human recombinant epidermal growth factor (EGF, Sigma-Aldrich) and TKI erlotinib hydrochloride (Santa Cruz Biotechnology) were used in the cell number experiments.

*Gene silencing by small-interfering RNA*

Small interfering RNA (siRNA) technology was used to silence CD151 gene expression in cell culture experiments. Two commercially available siRNA kits, purchased from Dharmacon (CO, USA) and Qiagen (Germany), were used. The CD151-specific-siRNA smartpool from Dharmacon comprised of four target sequences, GAUCAUCGCUGGUAUCCUC, GCAAGACGGUGGUGGCUCU, CCUCAAGAGUGACUACAUC and UCACAGGACUGGCGAGACA. The transfection control (trans ctrl) contained only DharmaFECT1 Transfection Reagent, whereas the negative vehicle control comprised the ON-TARGETplus Non-Targeting (NT) pooled siRNA. Secondly, the CD151 FlexiTube siRNA6 was purchased from Qiagen (SI02777257), which contained functionally verified siRNA directed against human CD151 (NM_001039490, NM_004357, NM_139029, NM_139030). The transfection control contained only HiPerFect Transfection Reagent, whereas the negative vehicle control also contained the ON-TARGETplus Non-targeting siRNA #3.

*Generation of pEGFP-CD151(CD151-overexpressing) cell lines*

The pEGFP-CD151 plasmid was generated by inserting the CD151 gene into the Hind-3 and Apa-1 sites of the multiple cloning sites of the pEGFP-N3 vector. The dose of 1.2μg/μl of geneticin (Life Technologies, CA, USA) was selected based as the killing concentration, whereas 0.6μg/μl geneticin was selected as the maintenance concentration for the stable cell lines.

The pEGFP-N3 and pEGFP-CD151 vectors were transfected at 5μg into A549 cells using 20μl of Lipofectamine LTX and 5μl of PLUS reagent (Invitrogen, MA, USA) for a 100mm culture dish, according to manufacturer’s instructions. Three stable clonal cell lines per expression plasmid, selected using 1.2μg/μl of geneticin, were verified and one clone was selected for all subsequent experiments. The stable cell lines, containing either pEGFP-N3 (vector control) or pEGFP-CD151 (CD151-overexpressing, CD151 OE), were grown and propagated in DMEM containing 0.4μg/μl geneticin, 10% v/v FBS, and 1% v/v P/S.
